# Strategic use of new generation antidepressants for depression: SUN(^_^)D study protocol

**DOI:** 10.1186/1745-6215-12-116

**Published:** 2011-05-11

**Authors:** Toshi A Furukawa, Tatsuo Akechi, Shinji Shimodera, Mitsuhiko Yamada, Kazuhira Miki, Norio Watanabe, Masatoshi Inagaki, Naohiro Yonemoto

**Affiliations:** 1 Department of Health Promotion and Human Behavior, Kyoto University Graduate School of Medicine/School of Public Health, Yoshida Konoe-cho, Sakyo-ku, Kyoto 606-8501 Japan; 2 Department of Psychiatry and Cognitive-Behavioral Medicine, Nagoya City University Graduate School of Medical Sciences, Mizuho-cho, Mizuho-ku, Nagoya 467-8601 Japan; 3 Department of Neuropsychiatry, Kochi Medical School, Kohasu, Okoh-cho, Nankokushi, Kochi 783-8505 Japan; 4 Department of Neuropsychopharmacology, National Institute of Mental Health, National Center of Neurology and Psychiatry, 4-1-1 Ogawa-Higashi, Kodaira, Tokyo 187-8553 Japan; 5 Department of Epidemiology and Biostatistics, Translational Medical Center, National Center of Mental Health, National Centre of Neurology and Psychiatry, 4-1-1 Ogawa-Higashi, Kodaira, Tokyo 187-8502 Japan; 6 Miki Clinic, 1-1-3 Hiranuma, Nishi-ku, Yokohama 220-0023 Japan; 7 Center for Suicide Prevention, National Institute of Mental Health, National Center of Neurology and Psychiatry, 4-1-1 Ogawa-Higashi, Kodaira, Tokyo 187-8553 Japan

## Abstract

**Background:**

After more than half a century of modern psychopharmacology, with billions of dollars spent on antidepressants annually world-wide, we lack good evidence to guide our everyday decisions in conducting antidepressant treatment of patients with major depression. First we did not know which antidepressant to use as first line treatment. Second we do not know which dosage we should be aiming at with that antidepressant. Because more than half of the patients with major depression starting treatment do not remit after adequate trial with the first agent, they will need a second line treatment. Dose escalation, augmentation and switching are the three often recommended second line strategies but we do not know which is better than the others. Moreover, we do not know when to start considering this second line treatment.

The recently published multiple-treatments meta-analysis of 12 new generation antidepressants has provided some partial answers to the first question. Starting with these findings, this proposed trial aims to establish the optimum 1st line and 2nd line antidepressant treatment strategy among adult patients with a non-psychotic unipolar major depressive episode.

**Methods:**

SUN(^_^)D, the Strategic Use of New generation antidepressants for Depression, is an assessor-blinded, parallel-group, multi-centre randomised controlled trial. Step I is a cluster-randomised trial comparing titration up to the minimum vs maximum of the recommended dose range among patients starting with sertraline. The primary outcome is the change in the Patient Health Questionnaire (PHQ)-9 scores administered by a blinded rater via telephone at week 1 through 3. Step II is an individually randomised trial comparing staying on sertraline, augmentation of sertraline with mirtazapine, and switching to mirtazapine among patients who have not remitted on the first line treatment by week 3. The primary outcome is the change in the PHQ-9 scores at week 4 through 9. Step III represents a continuation phase to Steps I and II and aims to establish longer-term effectiveness and acceptability of the above-examined treatment strategies up to week 25. The trial is supported by the Grant-in-Aid by the Ministry of Health, Labour and Welfare, Japan.

**Discussion:**

SUN(^_^)D promises to be a pragmatic large trial to answer important clinical questions that every clinician treating patients with major depression faces in his/her daily practices concerning its first- and second-line treatments.

**Trial registration:**

ClinicalTrials.gov: NCT01109693

## Background

### Depression is costly

Major depression is the 1^st ^leading cause of disability adjusted life years (DALY) lost excluding death, and the 3^rd ^leading cause of DALY including death in the world according to the most recent WHO estimates [1]. Moreover, this burden is expected to rise in the next 20 years. According to the same estimates, major depression is currently the 1^st ^leading cause of DALY excluding death and the 2^nd ^leading cause of DALY including death after cerebrovascular disease in Japan, comprising approximately 6% of all DALY lost among its people.

Major depression is indeed one of the most prevalent mental disorders in the United States and Europe, with 16.2% and 6.6% lifetime prevalence for American women and men [2] and with 16.5% and 8.9% for European women and men [3]. In Japan, while the point estimates are lower than in US or Europe, it is still the most prevalent mental disorder for its people, affecting one in 12 women (8.5%) and one in 29 men (3.5%) at least once in their lifetime [4].

Both pharmacotherapy and psychotherapy have been found to be equally effective in treating major depression [5] but the former remains the mainstay in everyday clinical practices due to its greater availability, tighter quality control and cheaper costs. Effective antidepressive agents include heterocyclic antidepressants (HCA), monoamine oxidase inhibitors (MAOI), selective serotonin reuptake inhibitors (SSRI), serotonin and noradrenaline reuptake inhibitors (SNRI), noradrenalinergic and specific serotonergic antidepressant (NaSSA) and others (such as bupropion). The dramatic rise in the consumption of antidepressants in developed countries in the past two decades has been mainly due to increase in use of SSRI, SNRI and other new generation antidepressants, which now are the most commonly prescribed antidepressants in the world [6]. In Japan the market for antidepressants had been hovering around 15 billion yen (166 million US dollars) per year up to 1999 but has been expanding by some 20% annually, reaching 120 billion yen (1.3 billion US dollars) in 2009, in which new generation antidepressants holds 89% share.

### Evidence on 1^st ^line choice of antidepressants

There is no question that we need a specific, detailed and appropriate guidelines in the treatment of major depression. However, all the guidelines up to 2008, including the one by the American Psychiatric Association [7], the one by the Canadian Psychiatric Association [8], the one by the National Institute of Clinical Excellence in the United Kingdom [9] and the Japanese one [10], recommend that the choice of antidepressants be made "on the basis of adverse effect profiles, cost, and patient preferences" [11] because there are differences in side effect profiles but not in effectiveness among various antidepressants [12].

However, in 2009, the research group from Japan, Italy and UK published the results of a systematic review of 117 RCTs (25928 subjects) of 12 new generation antidepressants in the acute phase treatment of major depression [13]. The Meta-analyses of New Generation Antidepressants (MANGA) study is based on the most comprehensive dataset of RCTs involving new generation antidepressants from the Cochrane Collaboration Depression, Anxiety and Neurosis Group and makes use of a new meta-analytic method called multiple-treatments meta-analysis (MTM; also sometimes referred to as network meta-analysis), which integrates data from direct (when treatments are compared within a randomised trial) and indirect comparisons (when treatments are compared between trials by combining results on how effective they are compared with a common comparator treatment). MTM thus allows a more precise estimate of comparative effectiveness with narrower confidence intervals than the traditional meta-analyses because it makes use of all direct and indirect comparisons. MTM also minimizes the influence of publication bias because a possible publication bias favoring a particular antidepressant can be counterbalanced by other similar biases favoring other antidepressants when all direct and indirect comparisons are combined through MTM.

The MANGA Study observed many statistically significant and clinical meaningful differences among the 12 new generation antidepressants. In terms of efficacy, mirtazapine, escitalopram, venlafaxine and sertraline were among the top four drugs; in terms of acceptability, escitaloporam, sertraline, bupropion and citalopram were superior to the others. The authors concluded that sertraline might be the best choice when starting treatment for moderate to severe major depression in adults because it has the most favorable balance between benefits, acceptability, and acquisition cost.

### Evidence on 2^nd ^line choice of antidepressants

Treatment of major depression is not easy because only some 50% respond, i.e. achieve depression severity less than half that at baseline, or only some 30% achieve remission, i.e. return to an euthymic state, after treatment with an adequate dose of antidepressant given for an adequate duration [14]. When patients show no to only partial response to the 1^st ^line treatment, 2^nd ^line treatments must be initiated. Guidelines recommendations for the 2^nd ^line treatment include dose escalation, switching to a different antidepressant possibly from a different class and augmentation [9,15]. Unfortunately, however, when many RCTs are planned and executed with the purpose of drug approval by the regulatory agency and as part of initial marketing strategy, evidence on the 2^nd ^line treatment is much scanter than that on the 1^st ^line.

First, with regard to dose escalation strategy, three systematic reviews have been published and all concluded that there is no evidence to suggest that dose escalation increases efficacy in comparison with continuing on the same dosage after failure to respond to the 1^st ^line antidepressant [16-18]. Next, with regard to switching, we find two systematic reviews in the literature [19,20] both of which was able to identify only one RCT that directly compared continuing on the same drug and switching to another. In this trial, 104 patients not responding to 6 weeks of fluoxetine 20 mg/d were randomly assigned to further 6 weeks of fluoxetine and switching to mianserin 60 mg/d; the remission rate was 18% and 36%, respectively (p = 0.10) [21]. When different switching options are compared, switching to venlafaxine after failure to respond on an SSRI may be marginally better than switching to another SSRI but there was no strong evidence to recommend other classes of antidepressants [20]. Lastly, many RCTs and systematic reviews have been published on various augmentation strategies. The ones with most randomized evidence include lithium augmentation [22], thyroid hormone augmentation [23] and augmentation with atypical antipsychotics [24]. Other options include augmentation with mirtazapine/mianserin [21,25,26] and augmentation with pindolol [27].

Even less evidence can be found comparing these different 2^nd ^line strategies against each other than comparing each strategy with staying on the former treatment. For example, the Sequenced Treatment Alternatives to Relieve Depression (STAR*D), which was funded by the NIMH and cost approximately 3 million dollars, examined five switching strategies and four augmentation strategies among the patients who had not achieved remission to the 1^st ^line SSRI treatment but was unable to compare switching versus augmentation as few patients agreed to this randomization [28,29].

### How to establish the optimum treatment strategy with new generation antidepressants

Review of the literature has revealed that there are indeed many urgent and critical clinical questions that must be answered before clinicians can confidently and competently administer pharmacotherapy for major depression. Urgent because every practitioner encounters these clinical questions almost on a daily basis. Critical because answers to these clinical questions can materially affect the patients' lives. Bandolier (http://www.medicine.ox.ac.uk/bandolier/index.html), an independent evidence review journal in UK, concluded its review on the MANGA Study by saying, "What the meta-analysis provides is the raw material for the next step, namely creating and testing a care pathway or pathways for depression that provides good results for the largest number of sufferers in the shortest time and at the lowest cost." (http://www.medicine.ox.ac.uk/bandolier/booth/mental/cipriani.html). This proposed study precisely aims to create and test this optimum care pathway for depression.

#### 1^st ^line treatment

According to the results of the MANGA Study, it is wise to use sertraline as 1^st ^line treatment of major depression in Japan because it represents the best balance in effectiveness and acceptability. However, practitioners immediately face an important clinical decision question at this stage, namely the problem of initial dosing strategy. The standard dosage range for sertraline is 50-100 mg/d but should clinicians aim at achieving 50 mg/d or 100 mg/d in the initial dosing strategy? Papakostas et al [30] published a systematic review of fixed-dose trials comparing different starting doses of SSRIs. In comparison with starting with the minimum of the standard dose range, starting with the maximum of the standard range may be more effective (RR = 1.12, 95%CI: 0.99 to 1.27) but less acceptable (0.74, 0.54 to 1.00). The response rate may increase from 51% to 54%, at the expense of the dropout rate also rising from 10% to 17%. It must be noted that they compared different starting doses, i.e. they administered the minimum or maximum of the standard dose range from the very beginning, and the dropouts are accounted for by last-observation-carried forward which is bound to affect and bias the results in an unknown way.

Can the initial dosing strategy to gradually increase the dosage up to the maximum of the standard range, recommended by many guidelines [8,10,31], be more effective and at least not any more unacceptable than the strategy to aim at the minimum of the standard range? No one knows the answer. It is truly unacceptable that a clinical question as urgent as this, because every single patient with major depression starting treatment with antidepressant faces this decision point, is not yet answered. We therefore planned an RCT to answer this question.

#### 2^nd ^line treatment

Even if we optimize the 1^st ^line antidepressant treatment strategy, more than half the patients cannot achieve remission [32]. What should we do as the 2^nd ^line treatment, and when should we make this decision?

No systematic review has found evidence for dose escalation and the present study will therefore not examine this option. There are many RCTs examining various augmentation strategies but only mirtazapine or mianserin augmentation is allowable according to the current Japanese regulations. As reviewed, we do not yet know which of augmentation or switching is superior in terms effectiveness and acceptability. Furthermore, we do not yet know when we should make this clinical decision to consider the 2^nd ^line treatment. Since each clinical research can answer only a limited number of well formulated clinical questions, this study will focus on switching to mirtazapine, which was the most effective antidepressant according to the MANGA study, and compare it to mirtazapine augmentation of SSRI, for which a number of RCTs provide some support.

Switching to mirtazapine is a plausible option as the 2^nd ^line treatment for the following reasons. (i) MANGA study showed mirtazapine may be the most effective new generation antidepressant. Due to its less favorable acceptability profile, it was not recommended as the 1^st ^line treatment but, when the latter fails, it is only logical to consider the more effective antidepressant. (ii) Switching is arguably to be preferred over augmentation because combining two drugs may lead to more known and unknown side effects than staying on the same drug.

Mirtazapine augmentation of SSRI is another option as the 2^nd ^line treatment for the following reasons. (i) A number of RCTs have provided some evidence to suggest its effectiveness. One small RCT randomly assigned 26 patients who had not responded to SSRI, bupropion or venlafaxine to augmentation either with mirtazapine 15-30 mg/d or with placebo. The remission rates were 46% versus 13% (p = 0.068) [26]. Another RCT administered fluoxetine plus mirtazapine or fluoxetine alone from the beginning of the acute phase treatment and the remission rates were 25% vs 52% (p = 0.052) [33]. (ii) It makes sense pharmacologically to combine sertraline, which is an SSRI (specific serotonin reuptake inhibitor), with mirtazapine, which is a NaSSA (noradrenergic specific serotonergic antidepressant). Mirtazapine increases noradrenaline and serotonin release through antagonism of central α_2_-adrenergic autoreceptors and heteroreceptors. Mirtazapine also exhibits antagonism to both 5-HT_2A_, 5-HT_2C _and 5-HT_3 _receptors, which results in a net increase in 5-HT_1_-mediated neurotransmission which is believed to be the primary mediator of efficacy of most antidepressant drugs. Antagonsim of the 5-HT_2A _receptors has beneficial effects on sexual dysfunction and insomnia, that of the 5-HT_2B _receptors on anxiety, and that of 5-HT_3 _on gastrointestinal symptoms, all of which constitute major side effects of SSRIs. (iii) Mirtazapine does not inhibit any liver enzymes and poses very low risk of interaction with other drugs. Sertraline exerts mild inhibition against CYP2D6 and 3A4 but is generally believed to be a safer drug when administered concomitantly with other drugs than many other SSRIs.

Another very important clinical question to be answered with regard to the 2^nd ^line treatment is when to consider it. As far as practitioners are concerned, this represents just as urgent a clinical question as that of initial titration strategy but, to the best of the authors' knowledge, no RCT has explicitly examined this issue and the guidelines are ambiguous and self-contradictory. For example, the guideline by the American College of Physicians [11] recommends that clinicians modify treatment if the patient does not have an adequate response to pharmacotherapy within 6 to 8 weeks of the initiation of therapy but this time frame appears to be based on the average length of clinical trials conducted mainly for drug approval. The NICE guidelines are self-contradictory as it recommends 3-4 weeks at one place and 6-8 weeks at another before considering the 2^nd ^line treatment alternatives [9]. We therefore decided to randomize the patients with regard to the 2^nd ^line treatment as early as 3 weeks and aimed to examine if considering the 2^nd ^line treatment at this early stage may or may not be beneficial in comparison with continuing the 1^st ^line treatment for 6 more weeks.

#### Continuation treatment

The last but not least factor to be considered in constructing the optimum treatment strategy for the 1^st ^and 2^nd ^line treatments is the continuation treatment following the acute phase treatment. A systematic review has unambiguously demonstrated that discontinuing antidepressants at the end of acute phase treatment can double the relapse/recurrence rates [34], and all the guidelines recommend continuation treatment of at least several months following acute phase treatment. However, in reality, many patients do not stay on the continuation phase [35]. It therefore follows that another very important factor in deciding the 1^st ^and 2^nd ^line treatment strategies is how easy and acceptable it is for patients to continue into the continuation treatment after acute phase treatment, in addition to their effectiveness and acceptability during the acute phase treatment.

### Aims

The current randomized trial aims to elucidate "pathways for depression that provides good results for the largest number of sufferers in the shortest time and at the lowest cost" (Bandolier 2009). More specifically, the objectives of this trial are to examine the following treatment options among patients with an untreated, non-psychotic unipolar major depressive episode:

(1) When the 1^st ^line treatment is started with sertraline, which is better as an initial prescription strategy up to 3 weeks in terms of effectiveness and safety (i.e. side effects and treatment continuation), to titrate to the lowest dosage of the effective range or to its highest dosage?

(2) When the patients do not remit on the 1^st ^line treatment at 3 weeks, which is better as acute phase treatment up to 9 weeks in terms of effectiveness and safety, to continue sertraline, to augment sertraline with mrtazapine or to switch to mirtazapine?

(3) Which of the above strategies of 1^st ^and 2^nd ^line treatments is better as acute phase and continuation treatments up to 25 weeks in terms of effectiveness and safety?

## Methods

This is an assessor-blinded, parallel-group, multi-centre randomized controlled trial.

### Participants

Participants will be recruited from among those visiting the clinical trial sites according to the following eligibility criteria.

#### Inclusion criteria

1) The participant fulfills criteria for non-psychotic unipolar major depressive episode (DSM-IV) within one month before starting sertraline

2) Age between 25 and 75 on the day when sertraline is started

3) The major depressive episode is the focus of the treatment and the treating physician has judged sertraline to be its appropriate 1^st ^line drug

4) Tolerability to sertaline has been ascertained after 3-16 days of treatment with sertraline 25 mg/d

5) The participant is able to understand and sign written informed consent

6) The participant is available on the phone for assessment of symptoms and side effects

#### Exclusion criteria

1) Having taken antidepressants, mood stabilizers (lithium, valproate, carbamazepine), antipsychotics, psychostimulants (methylphenidate, pemoline, atmoxetine), electroconvulsive therapy, or depression-specific psychotherapies (cognitive-behavior therapy, interpersonal therapy) within one month before starting sertraline

2) History of schizophrenia, schizoaffective disorder or bipolar disorder (DSM-IV) as judged by treating physician

3) Current dementia, borderline personality disorder, eating disorder or substance dependence (DSM-IV) as judged by treating physician

4) Physical diseases which may contraindicate treatment with sertraline or mirtazaapine

5) Allergy to sertraline or mirtazapine

6) Terminal physical diseases

7) Women who are pregnant or breastfeeding (if there is a possibility of getting pregnant within 6 months of trial entry, participation is allowed only after providing signed consent to avoid pregnancy during the trial period)

8) Imminent high risk of suicide as judged by treating physician

9) Needing non-voluntary hospitalization

10) High probability of changing hospital due to relocation etc within 6 months of trial entry

11) Cohabiting family members of research staff members of the trial

12) Inability to understand written Japanese

Nb

1) A comprehensive systematic review and meta-analysis has shown that antidepressants increase suicidality in comparison with placebo for people under age 25 but decreases suicidality for people aged 25 or older [36].

2) Both males and females are accepted.

3) There is no eligibility criteria for severity of depression as long as the participant meets the diagnostic criteria for major depression. Both outpatients and inpatients are accepted.

4) Patients having taken benzodiazepine anxiolytics, tandospirone, hydroxyzine, hypnotic medications, traditional Kampo medications within one month before starting sertraline are not excluded.

5) Patients having received psychotherapies other than depression-specific ones (cognitive-behavior therapy and interpersonal therapy) are not excluded.

6) Patients with physical diseases that the treating physician judged would not interfere with treatment with sertraline or mirtazapine are not excluded.

7) The participant will continue the trial even if his/her diagnosis is changed after trial entry.

### Trial Site Recruitment

#### Eligibility criteria for a trial site

A participating trial site must fulfill the following eligibility criteria.

1) It must have a department of psychiatry or of psychosomatic medicine.

2) The principal trial physician and all the participating trial physicians at the site must have understood the study protocol (e.g. cluster randomization to 50 mg/d or 100 mg/d of sertraline at Step I) and have agreed to collaborate.

Nb

A site-visiting CRC will be dispatched to a trial site which

1) Is located within one hour at most approximately from the regional centre

2) Has more than 100 first-visit patients with major depression per annum

3) Has a separate room that the CRC can use for informed consent and that the central assessor can use for telephone assessment.

Such trial sites will open, if possible, "a trial clinic" on a certain day of the week to facilitate patients' participation.

#### Procedure for a trial site to participate

Each regional centre will recruit collaborating trial sites (psychiatric private practice, department of psychiatry of a general hospital, psychiatric hospital) in units of 4-5.

If the trial site has its own Institutional Review Board, the principal trial physician will seek approval from his/her own IRB and then fax the document of approval to the national centre office. The national central office will examine the document(s) and return the review results to the trial site principal physician by email.

If the trial site does not have its own IRB, the principal trial physician will send a proxy form to the IRB at its regional centre and seek approval there.

Before the trial site starts recruiting the participants, all the principal trial physician and the participating trial physicians must attend the start-up meeting held either at the trial site or at the national centre. The co-PI and CRC at the regional centre will visit each trial site in order to make sure that the site has finished the preparation and to rehearse the EDC system and blinded central telephone assessment.

### Procedures

**Figure 1:**
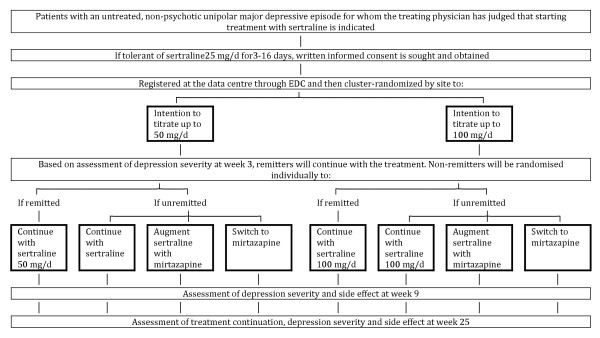
Flow diagram of the trial

**Figure 2:**
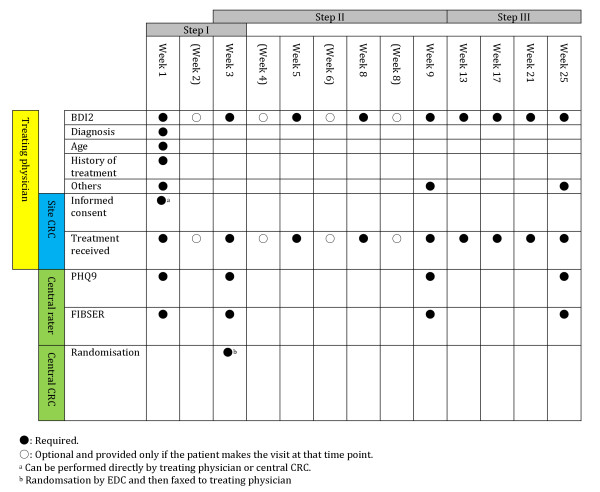
Schedule of the planned assessments for Steps I, II and III

The overall procedure of the trial is shown in Figures 1 and 2.

#### Formulation of clinical questions

Clinical questions to be answered at each step can be formulated as follows.

##### Step I

Patients: Patients with non-psychotic unipolar major depressive episode who had not received treatment for the index episode before starting sertraline and who tolerate sertraline 25 mg/d

Exposure1: Strategy to titrate sertraline up to the maximum of the effective range, i.e. 25 mg/d -> 50 mg/d -> 100 mg/d

Exposure2: Strategy to titrate sertraline upt to the minimum of the effective range, i.e. 25 mg/d -> 50 mg/d -> 50 mg/d

Outcomes: The primary outcome is the change inPHQ9 scores at week 1 through week 3

The secondary outcomes include:

1) Change in BDI2 scores at week 1 through week 3

2) Proportion of remission (4 or less on PHQ9) at week 3

3) Proportion of response (50% or greater reduction on PHQ9) at week 3

4) Proportion of successful continuation of the allocated treatment up to week 3

5) Change in FIBSER at week 1 through week 3

6) Change inPHQ9 at week1 through week 9

7) Change in BDI2 at week 1 through week 9

8) Proportion of remission (4 or less on PHQ9) at week 9

9) Proportion of response (50% or greater reduction on PHQ9) at week 9

10) Proportion of successful continuation of the allocated treatment up to week 9

11) Change in FIBSER at week 1 through week 9

12) Suicidality as assessed with C-CASA between week 1 and week 9

13) Manic/hypomanic/mixed episode between week 1 and week 9

14) Serious adverse events between week 1 and week 9

##### Step II

Patients: Patients whose major depressive episode did not remit (5 or more on PHQ9) at week 3 to the 1^st ^line treatment with sertraline

Exposure1: Continue sertraline 50 mg/d or 100 mg/d for 6 more weeks

Exposure2: Augment sertraline with mirtazapine 15-45 mg/d

Exposure3: Switch to mirtazapine 15-45 mg/d

Outcome: The primary outcome is the change in PHQ9 at week4 through week 9

The secondary outcomes include:

1) Change in BDI2 at week 4 through week 9

2) Proportion of remission (4 or less on PHQ9) at week 9

3) Proportion of response (50% or greater reduction on PHQ9) at week 9

4) Proportion of successful continuation of the allocated treatment up to week 9

5) Change in FIBSER at week 4 through week 9

6) Suicidality as assessed with C-CASA between week 3 and week 9

7) Manic/hypomanic/mixed episode between week 3 and week 9

8) Serious adverse events between week 3 and week 9

##### Step IIIa [exploratory analysis of continuation treatment for Step I]

Patients: Patients with non-psychotic unipolar major depressive episode who had not received treatment for the index episode before starting sertraline and who tolerate sertraline 25 mg/d

Exposure1: Strategy to titrate sertraline up to the maximum of the effective range, i.e. 25 mg/d -> 50 mg/d -> 100 mg/d by week 3, then allocated to continue sertraline between week 3 and week 9, then treated at the discretion of the trial physician

Exposure2: Strategy to titrate sertraline up to the minimum of the effective range, i.e. 25 mg/d -> 50 mg/d -> 50 mg/d by week 3, then allocated to continue sertraline between week 3 and week 9, then treated at the discretion of the trial physician

Outcome: The primary outcome is the proportion of patients who continue the allocated treatment up to week 25 and are in remission (4 or less on PHQ9) at week 25

The secondary outcomes include:

1) Proportion of patients who continue the allocated treatment up to week 25 and are showing response (50% or greater reduction on PHQ9) at week 25

2) Rate of continuation of allocated treatments up to week 25

3) Change in PHQ9 at week 1 through week 25

4) Change in BDI2 at week 1 through week 25

5) Suicidality as assessed with C-CASA between week 1 and week 25

6) Manic/hypomanic/mixed episode between week 1 and week 25

7) Serious adverse events between week 1 and week 25

##### Step IIIb [exploratory analysis of continuation treatment for Step II]

Patients: Patients whose major depressive episode did not remit (5 or more on PHQ9) at week 3 to the 1^st ^line treatment with sertraline

Exposure1: Continue sertraline 50 mg/d or 100 mg/d for 6 more weeks, then treated at the discretion of the trial physician

Exposure2: Augment sertraline with mirtazapine 15-45 mg/d up to week 9, then treated at the discretion of the trial physician

Exposure3: Switch to mirtazapine 15-45 mg/d up to week 9, then treated at the discretion of the trial physician

Outcome: The primary outcome is the proportion of patients who continue the allocated treatment up to week 25 and are in remission (4 or less on PHQ9) at week 25

The secondary outcomes include:

1) Proportion of patients who continue the allocated treatment up to week 25 and are showing response (50% or greater reduction on PHQ9) at week 25

2) Rate of continuation of allocated treatments up to week 25

3) Change in PHQ9 at week 4 through week 25

4) Change in BDI2 at week 4 through week 25

5) Suicidality as assessed with C-CASA between week 3 and week 25

6) Manic/hypomanic/mixed episode between week 3 and week 25

7) Serious adverse events between week 3 and week 25

#### Pilot study

In order to test the feasibility of the study, a pilot study will be run according to this same protocol between December 2010 and October 2011. The pilot study will be a multi-centre study involving:

• Nagoya City University Hospital and its affiliated private practices and departments of psychiatry in a general hospital

• Kochi Medical School Hospital and its affiliated private practices, departments of psychiatry in a general hospital and psychiatric hospitals

• Private practices in Yokohama

The Nagoya site will test recruitment using site CRCs, the Kochi site will test recruitment using site CRCs and direct recruitment by trial physicians, and the Yokohama site will test recruitment using site CRCs dispatched from a commercial site management organization. Feasibility and efficiency of these different recruitment methods will be examined.

The pilot study will use data of the 1^st ^200 patients up to week 25. The pilot study will be reviewed by DSMB who will advise the Steering Committee on the feasibility and safety of the study and on appropriateness of continuing the study. The final decision about whether to continue the study will be made by the Steering Committee. Before continuing the study, the protocol may be amended if necessary and additional trial sites will be recruited.

#### Step I

##### Ascertaining eligibility criteria

The trial physician and/or site CRC will seek informed consent from a participant at week 1, i.e. 3-16 days after starting sertraline 25 mg/d. The "3-16 days" time frame was chosen to allow two possible visit days to accommodate the participant's schedule at a site where the site CRC makes his/her visits every week. After obtaining the written informed consent, the trial physician or the site CRC makes a face-to-face interview or the central CRC or the central rater will make a telephone interview to assess

1) PHQ9 at week 1

2) FIBSER at week 1

These week 1 assessment results will be entered into the EDC along with the complete data on the "Eligibility Form."

##### Allocation to treatments

Eligible participants will be allocated 1:1 to the sertraline 50 mg/d arm and to the sertraline 100 mg/d arm. We will employ cluster randomization by trial site. This cluster randomization will be made by the EDC system. The allocation will use the minimization method adjusting for the number of probable entries as judged by the principal investigator and co-principal investigators (40 or more participants per year vs less than 40 participants per year).

We employ cluster randomization for Step I for the following reasons.

1) The comparison for Step I is between physician's choice of a strategy to titrate sertraline used as the 1^st ^line antidepressant up to the minimum effective range or up to the maximum effective range. It is therefore logical to randomize by physician.

2) In reality, because this is an open trial in which the trial physician gradually titrates the dosage taking into account the side effects, having one patient in the sertraline 100 mg/d arm and another in the sertraline 50 mg/d arm may at the same time create contamination in the doctor's decisions. That is, if we randomized by patient, the doctor might tend to stick to his/her personally preferred titration schedule regardless of the individual patient's assignment and reported side effects.

3) Likewise, having different doctors with different titration policies within the same trial site might cause unnecessary confusion among the physicians and co-medical staff at the site.

4) Asking the participant to undergo individual randomization twice might increase the barrier to participation.

5) A number of previous studies have repeatedly reported negligible to very small intra-cluster correlation coefficients [37,38].

##### Treatments

The trial physician will prescribe according to either of the following schedule, depending on his/her own allocated treatment strategy.

1) In the 100 mg/d arm, prescribe 50-75 mg/d (once after dinner or before bedtime) for one week at week 1, then prescribe 100 mg/d (once or divided twice per day) for one week at week 2

2) In the 50 mg/d arm, prescribe 50 mg/d (once after dinner or before bedtime) for one week at week 1, then prescribe the same regimen for one week at week 2

##### Outcome assessments

The trial physician or the site CRC will ask the participant to fill in BDI2 upon week 2 and week 3 visits. The CRC or the physician will enter the data into EDC.

At week 3, the central rater will administer

1) PHQ9 at week 3

2) FIBSER at week 3

by telephone. The central CRC will obtain the patient's name and phone number and will keep the rater blind to the name of the clinic and the treatment the participant is receiving. This telephone assessment will normally be conducted in a separate room after the patient arrives at the clinic and before the consultation with the trial physician, so that imminent suicidality may be handled promptly and appropriately according to the "Suicidality Management Manual." If the patient has dropped from the treatment, the telephone call will be made to the mobile phone which he/she has previously registered upon entry into the trial. If strong suicidal wishes are expressed, the central rater will follow the "Suicidality Management Manual."

#### Step II

##### Ascertaining eligibility criteria

If the patient scores 5 or more on PHQ9 at week 3, as assessed by the central rater, he/she will be randomized for Step II according to the following procedures.

If the patient scores 4 or less on PHQ9 at week 3, he/she will continue on the same regimen, and receive the assessments at week 9 and week 25 as planned.

##### Allocation to treatments

The patients scoring 5 or more on PHQ9 at week 3 will be allocated 1:1:1 to the continue-sertraline arm, the mirtazapine augmentation arm, and the mirtazapine switch arm. This randomization will use the minimization method adjusting for (i) site, (ii) whether 50% or greater reduction on PHQ9 is achieved or not, and (iii) whether "moderate" or greater impairment due to side effects is reported on item 4 of FIBSER.

The central CRC will enter the necessary data from PHQ9 and FIBSER into EDC. The EDC program will then output "The patient is making steady recovery. Please continue with the same regimen" if the PHQ9 score is 4 or less, and any one of "Augment with mirtazapine. Please add mirtazapine 15 mg/d," "Switch to mirtazapine. Decrease sertraline to half the current dose and add mirtazapine 15 mg/d," or "Continue with sertraline" if thePHQ9 score is 5 or more according to the above randomization. The central CRC will fax the output to the trial physician and the site CRC, so that the physician need not start up the computer every time.

If the EDC server is down and/or the trial site cannot use the EDC system for various reasons, the randomisation can be done by calling up the central CRC or the data centre.

##### Treatments

The details of the three intervention arms are as follows.

1) Continue sertraline as specified by Step I cluster randomization. Between week 4 and week 9, sertraline must be kept within the maximum as specified by Step I cluster randomization. If the dosage has not reached this maximum (e.g. 100 mg/d) at week 3, it can be increased to this maximum (in this case, 100 mg/d) between week 3 and week 9.

2) Continue sertraline as specified by Step I cluster randomization and add mirtazapine 15 mg/d at bedtime to augment sertraline. After week 4, mirtazapine can be given in 7.5-45 mg/d at bedtime. Between week 4 and week 9, sertraline must be kept within the maximum as specified by Step I cluster randomization. If the sertraline dosage has not reached this maximum (e.g. 100 mg/d) at week 3, it can be increased to this maximum (in this case, 100 mg/d) between week 3 and week 9. Mirtazapine should usually be started at 15 mg/d but can be halved by the treating psychiatrist taking into account age etc of the patient.

3) Decrease sertraline to half the current dose and add mirtazapine 7.5-15 mg/d at bedtime in order to switch to mirtazapine. Sertraline must be halved at week 3 and stopped by week 4 or week 5 (sertraline should no longer be prescribed at week 7 at the latest), so that the patient will receive mirtazapine 7.5-45 mg/d only between week 7 and week 9.

##### Outcome assessments

The trial physician or the site CRC will continue to ask the participant to fill in BDI2 at every visit between week 4 and week 9. The CRC or the physician will enter the data into EDC.

At week 9, the central rater will administer

1) PHQ9 at week 9

2) FIBSER at week 9

by telephone. The central CRC will obtain the patient's name and phone number and will keep the rater blind to the name of the clinic and the treatment the participant is receiving. This telephone assessment will normally be conducted in a separate room after the patient arrives at the clinic and before the consultation with the trial physician, so that imminent suicidality may be handled promptly and appropriately according to the "Suicidality Management Manual." If the patient has dropped from the treatment, the telephone call will be made to the mobile phone which he/she has previously registered upon entry into the trial. If strong suicidal wishes are expressed, the central rater will follow the "Suicidality Management Manual."

#### Step III

##### Ascertaining eligibility criteria

All the participants who have entered the trial are eligible.

##### Treatments

All the available treatment guidelines for depression recommends that the acute phase treatment, if successful, be continued at least several months. All the treatments between week 9 and week 25 are at the treating physician's discretion. He/she may continue with the same regimen or completely change the regimen. Electroconvulsive therapy and depression-specific psychotherapies can also be administered.

##### Outcome assessments

The trial physician or the site CRC will continue to ask the participant to fill in BDI2 at every visit between week 10 and week 25. The physician or the CRC will enter the data into EDC.

At week 25, i.e. approximately 6 months after trial entry and 4 months after week 9 assessments, the central rater will administer

1) PHQ9 at week 25

2) FIBSER at week 25

3) History of prescription up to week 25, especially how long the treatment assigned at week 3 was adhered to

by telephone. The central CRC will obtain the patient's name and phone number and will keep the central rater blind to the name of the clinic and the treatment the participant is receiving. This telephone assessment will normally be conducted in a separate room after the patient arrives at the clinic and before the consultation with the trial physician, so that imminent suicidality may be handled promptly and appropriately according to the "Suicidality Management Manual." If the patient has dropped from the treatment, the telephone call will be made to the mobile phone which he/she has previously registered upon entry into the trial. If strong suicidal wishes are expressed, the central rater will follow the "Suicidality Management Manual."

### Concurrent Treatments

#### Permitted concurrent treatments

The following medications are allowed throughout the trial at the discretion of the trial physician.

1) Benzodiazepine anxiolytics and hypnotics

2) Tandospirone, hydroxyzine

3) Gastrointestinal and digestive drugs (except for sulpiride)

4) Medications for concurrent physical diseases

5) Non-specific psychotherapies (psychotherapies other than depression-specific CBT and IPT), exercise therapy, music therapy, family psychoeducation

#### Prohibited concurrent treatments

Through Step I and Step II, the following treatments are prohibited in principle. However, the patient's safety should be the utmost concern and takes priority over everything else, all appropriate care should be given depending on the patient's condition.

1) Antidepressants other than sertraline or mirtazapine

2) Antipsychotics

3) Mood stabilizers (lithium, valproate, carbamazepine)

4) Depression-specific psychotherapies (CBT, IPT)

5) Electroconvulsive therapy

There is no prohibited treatments for Step III.

### Stopping Rules For Participants & Trial Sites

#### Deviation from protocol treatment

The following cases will be considered deviation from the trial protocol. The participant, however, will not be considered to have dropped out of the study at this stage and will receive the protocol assessments.

1) Prohibited concurrent treatments were administered in Step I or II

2) The participant was not randomised within the pre-specified time frame for week 3.

3) The participant cannot take any sertraline in Step I.

4) The participant switches to manic/hypomanic/mixed in Step I.

5) The participant turns out to suffer from bipolar disorder, schizophrenia or dementia in Step II.

The treating physician is to judge whether 1) through 5) has occurred. If so judged, the physician should immediately notify the site CRC, who will notify the central office.

If 1) through 4) occurs in Step I, the randomization for step II will not be made but assessment will continue.

If 1) or 5) occurs in Step II, the patient will be analysed as randomized.

#### Stopping intervention

If the participant meets any one of the following conditions, the trial physician will stop the protocol treatment at his/her discretion. The participant, however, will not be considered to have dropped out of the study at this stage and will receive the protocol assessments.

1) The participant wishes to stop the protocol treatment.

2) The trial physician judges that it is difficult to continue the protocol treatment because of emergence of serious adverse events (SAE) as defined below.

3) The trial physician judges that the risk outweighs benefit in continuing the protocol treatment even when no SAE is reported.

4) The participant becomes pregnant and the trial physician judges that the risk outweighs benefit in continuing the protocol treatment.

5) The trial physician judges that it is inappropriate to continue the protocol treatment for any other reason.

#### Stopping assessment

If the participant meets any one of the following conditions, he/she will never be contacted for assessments.

1) The participant withdraws consent to receiving protocol assessments, regardless of whether he/she is continuing the protocol treatment.

#### Dropping trial sites

If the trial site meets any of the following conditions, it will be judged "dropout" and will no longer be able to recruit patients. However, the patients who have already entered the study will be followed-up.

1) The principal trial physician withdraws his consent.

2) No study entry was made within 6 months.

3) The Steering Committee judges that it is inappropriate to continue recruitment at this site.

### Assessments

#### Measures

##### Patient Health Questionnaire-9 (PHQ9)

The Patient Health Questionnaire was developed in 1999 as a self-report version of the Primary Care Evaluation of Mental Disorders (PRIME-MD) which aims at criteria-based diagnosis of several mental disorders commonly seen in primary care [39]. The depression module of the PHQ is called PHQ9 and consists of the nine diagnostic criteria items of the DSM-IV. Each item is rated between 0 = "Not at all" through 3 = "Nearly every day," making the total score range between 0-27. Excellent test-retest reliability (ICC = 0.92) [40] and internal consistency reliability (Cronbach's alpha = 0.89) [39] have been reported. Good construct validity has been demonstrated through associations with various severity indices [41]. The sensitivity to change is as good as or better than extant scales [42].

Kroenke and his colleagues have provided the following rules of thumb for interpreting the continuous PHQ9 scores [41].

0-4 no depression

5-9 mild depression

10-14 moderate depression

15-19 moderately severe depression

20- severe depression

The minimal clinically important difference, i.e. the smallest difference in score that is considered to be a clinically important intra-individual change, was established to be 5 [42].

The PHQ9 should require less than one minute to fill in for the patient and less than one minute to administer for the clinician [41]. The Japanese version has been established by Muramatsu through backtranslation [43].

In this trial, PHQ9 will be administered 5 times at week1, week 3, week 9 and week 25. The central rater will receive training in administering PHQ9 through simulated interviews and will have demonstrated satisfactory reliability. The blindness of the central rater as to the participant's treatment will be assessed by asking the central rater to guess the allocated treatment at week 3, 9 and 25 assessments.

##### Beck Depression Inventory-II (BDI2)

BDI2 is a 21-item self-report instrument to measure the severity of depression in adolescents and adults. Its first version was developed in 1961 and slightly amended in 1979 but in 1996 a major revision was undertaken to make the scale more congruent with the modern diagnostic criteria for major depression. In its 40 years of usage, the BDI has become one of the most widely used instruments for detecting possible depression in normal populations and for assessing severity of depression in diagnosed patients [44].

The time frame for evaluation is set to the past 2 weeks including the day of assessment. Each item in the BDI2 has a series of four statements, which describe symptom severity along an ordinal continuum from absent or mild (a score of 0) to severe (a score of 3). The total score therefore ranges from 0 through 63.

Good reliability and validity have been reported for the original [45] as well as the Japanese version [46].

The original authors proposed the following rules of thumb for interpreting the BDI2 scores [45]

0-13 Minimal

14-19 Mild

20-28 Moderate

29-63 Severe

Two subsequent studies from the US and from Japan basically confirmed these interpretations [46,47].

A rough guide for interpreting the changes in BDI2 scores may be [46]

0-9 no or slight change, with 5 indicating a minimally important clinical difference

10-19 moderate change

>= 20 large change.

Most patients are comfortable with this 21-item questionnaire and can complete them within 5-10 minutes.

In this trial, BDI2 will be filled in by the patient at each visit.

##### Frequency, Intensity, and Burden of Side Effects Rating (FIBSER)

FIBSER was originally used in STAR*D as a global rating scale for side effects. This is an observer-rated scale and the Japanese translation has not gone through backtranslation.

##### Continuation of protocol treatment

Continuation of protocol treatment without stopping intervention or stopping assessment as defined above is called "treatment continuation." In Step III, concurrent treatments prohibited in Steps I and II can be used and this will not constitute "treatment continuation."

#### Timing of assessments

Assessments at week 3, 9 and 25 may be made within the following time frames after week 1.

1) ±4 days for assessments at week 1 through 9

2) ±14 days for assessments after week 9

Assessment for week 3 must be made between -4 days to +14 days of the planned date, and that for week 9 must also be made between -4 days to +14 days of the planned date.

#### Data monitoring and site audit

##### Data monitoring

The data centre will provide the following data monitoring report to the Steering Committee and the DSMB every six months. The chair of the DSMB will assess the data monitoring report, and if he/she finds an ethical problem in the continuation of the trial from the viewpoints of safety or effectiveness, he/she will convene the DSMB and advise the principal investigator to change or stop the study.

The data monitoring report will include:

1) Progress of the trial regarding trial entry and follow-up

2) Implementation of assessments (allocation will be masked)

3) Incidence of serious adverse events (allocation will be masked)

4) Any other problems.

##### Site audit

Each site will be surveyed within 6 months after the study commencement. The site audit team nominated by the Steering Committee will survey the sites according to the Site Audit Manual. The audit team will report the results to the Steering Committee, which will review them.

### Reporting of adverse events and protection of participants

#### Definition of adverse events

An adverse event is defined as any unwanted or unintended sign (including laboratory exams), symptom or disease seen in participants of the trial, regardless of the causal relationship with the study intervention.

#### Reportings according to Pharmaceutical Affairs Act (1950 Law 145)

All the protocol interventions in the current trial are within the approved dosage and administration in Japan and will therefore have to follow the Japanese Pharmaceutical Affairs Act.

Adverse events will be assessed according to the "Adverse Events Manual" which follows the Japanese Ministry of Health, Labour and Welfare's "Manual for rating the severity of side effects by pharmaceutical products," with an amendment to allow more detailed assessment of suicidality according to Columbia Classification Algorithm for Suicide Assessment (C-CASA) [48].

Adverse events will be classified into:

Grade 1: minor side effects

Grade 2: neither major nor minor side effects

Grade 3: major side effects, i.e. side effects that may lead to death or to enduring severe impairment depending on the patient's conditions and circumstances

All grade 3, and unforeseeable grade 2 adverse events shall be reported to the relevant section of the Ministry of Health, Labour and Welfare as well as to the national centre office. Foreseeable adverse events are judged according to the package inserts of respective drugs. Any grade 3 adverse events that occurred within 30 days of the completion of the protocol treatment shall be reported to the Ministry and the national centre office. The reporting shall be done using the attached "Reporting form on safety of pharmaceutical products."

The principal investigator, upon receiving the report, will consult with the trial physician to discuss the course of actions to be taken with regard to the patient in question and also with regard to the study.

#### Reportings according to the Ethics Guideline for Clinical Research (Ministry of Health, Labour and Welfare, revised in 2008)

When a serious adverse event occurs, the trial physician must take all the necessary and appropriate measures to ensure safety of the participant. He/she must also notify the principal investigator immediately. The principal investigator must notify co-principal investigators at all the regional centres within 24 hours, and report to the head of the clinical research institution (In the pilot study, Director of Nagoya City University Hospital or Director of Kochi Medical School Hospital) through the co-principal investigators. The principal investigator must also notify all the collaborators. The head of the clinical research institution must report to its own IRB and, if it concerns an unforeseeable serious adverse event, must report to the Ministry of Health, Labour and Welfare.

"A serious adverse event" is defined here as "an adverse event that may lead to death or to enduring severe impairment depending on the patient's conditions and circumstances" and will include:

1. Death

1.1. All deaths regardless of causal relationship with the protocol treatment, if it is a death during the protocol treatment

1.2. Deaths whose causal relationship with the protocol treatment cannot be denied, if it is a death within 30 days after completion of the protocol treatment.

2. Life-threatening event

3. Event leading to enduring and severe impairment and dysfunction

When treatment is required, the trial physician will provide and/or arrange appropriate treatments including hospital admission.

#### Foreseeable adverse events according to the package inserts

##### Sertraline

Frequent side effects

Nausea (18.9%), somnolence (15.2%), dry mouth (9.3%), headache (7.8%), diarrhea (6.4%), dizziness (5.0%) etc.

Serious side effects

Serotonin syndrome (unknown frequency), malignant syndrome (unknown frequency), convulsion (unknown frequency), coma (unknown frequency), liver dysfunction (unknown frequency), SIADH (unknown frequency), Lyell syndrome & toxic epidermal necrolysis (unknown frequency), anaphylactoid symptoms (unknown frequency)

##### Mirtazapine

Frequent side effects

Somnolence (50.0%), dry mouth (20.6%), fatigue (15.2%), constipation (12.7%), increased AST/ALT (12.4%)

Serious side effects

Serotonin syndrome (unknown frequency), agranulocytosis/neutropenia (unknown frequency), convulsion (unknown frequency), liver dysfunction/jaundice (unknown frequency), SIADH (unknown frequency)

### Stopping Rule For Study

The study will be discontinued by the Steering Committee (or the principal investigator in the case of an emergency) upon advice from the DSMB if any of the following conditions is met.

1) The causal relationship between any of the protocol treatments and serious adverse events including death is established by this study or by any other study.

2) Provision of study drugs becomes impossible for any reason.

### Data Management And Publication Policy

#### Data Management

The data management will be done by the data centre. The electronic data is anonymized in a linkable record, and the participants' names and ID numbers will be recorded only on non-electronic media (e.g. paper notebook) and kept at each trial site.

The central CRC will check the progress of all the entered participants every day by use of the EDC and will contact the site CRC or the trial physician should any doubt arise.

The data centre will perform similar checks and will contact the central CRC should any doubt arise.

#### Publication Policy

The protocol will be published, with TAF as first author. 

The main papers stemming from Steps I, II and III, especially the one from Step II, will be submitted to a high impact journal. The collaborating researcher has the right to be the first author of these papers in order of their number of recruitment. TAF will remain the corresponding author for all the papers. All the trial principal physicians and the trial participating physicians who have entered more than 10 patients will appear as co-author of at least one paper. 

Trial principal physicians, trial participating physicians and other members of the Steering Committee, if they do not appear as co-author, will be listed at the end of the article. Such authors may be counted as co-authors in some journals but not in others. 

The results shall be reflected in treatment guidelines and systematic reviews.

### Study Period

The study period of this trial will be between December 2010 and March 2014, with the patient entry period between December 2010 and September 2013.

The study period of the pilot study will be between December 2010 and March 2012, with the patient entry period between December 2010 and October 2011.

### Statistical Analyses

#### Sample size calculation

##### Sample size for Step I

Assuming an intra-cluster correlation coefficient to be 0.05 [37,38], with alpha error at 0.05 and statistical power at 0.80, to detect a mean difference of 1 point on PHQ9 (SD = 5), i.e. to detect an effect size of 0.2, we need 66 patients at each of 30 sites. The total sample size is therefore 1980.

##### Sample size for Step II

The clinical question for Step II is the main hypothesis of this trial. Previous studies using PHQ9 in the acute phase treatment of major depression have shown that, on average, the PHQ9 scores will drop from 15 (SD = 5) at baseline to 10 (SD = 6) at end of treatment, with a mean change of 5 (SD = 5) [49-51]. We expect a difference of 20% (1 point) in the PHQ9 change scores among the intervention arms and consider this to be a clinically meaningful difference in effect. With alpha error set at 0.05 and statistical power at 0.80, in order to detect a between-group difference of 1 point (SD = 5) in the reduction of PHQ9 scores from baseline, we need 522 per group and 1566 in toto at Step II. Assuming a dropout rate of 20% and a remission rate of 10% at week 3, we need 2175 participants for Step I.

One point difference in the mean change score on PHQ9 corresponds with an effect size of 0.2. This is a small effect according to Cohen's rough rule of thumb for effect size interpretation [52]. However, because the present trial represents comparison among active treatments and because the true effect size of antidepressants over placebo appears to be around 0.3 [53] and the average effect size of all the health interventions examined in the Cochrane Library appears to be between 0.3 and 0.4 [54], we consider this to be a clinically meaningful difference in effectiveness worth detecting in a large clinical trial. As a matter of fact, an effect size of 0.2 will be translated into an NNT of 10 if the control event rate is around 50% (e.g. response as defined usually by 50% or greater reduction in depression severity from baseline) and 20 if the control event rate is around 20% (e.g. remission of depression) [55]. They therefore represent clinically meaningful difference in effect.

The sample size will be revisited after completion of the pilot study.

##### Sample size for Step III

Step III represents continuation treatment for Steps I and II, and will therefore be examined as exploratory studies. We therefore will not calculate sample size necessary to detect a significant difference. However, we will calculate the obtained statistical power post-hoc.

##### Sample size for pilot study

The pilot study is a feasibility study and needs no sample size calculation. The target sample size is 200. We will perform no statistical analyses looking at the comparison arms at the end of the pilot study, whose participants will therefore be included in the main study unless there is a major change in the study protocol.

#### Statistical analyses

##### Primary analyses

For Step I, we will compare the sertraline 50 mg/d arm and the sertraline 100 mg/d arm at an individual level. We will test whether the changes in PHQ9 scores at week 1 through week 3 are statistically significantly different between the two arms. Because Step I employs cluster randomization, we will have to take into consideration intra-class correlation coefficients.

For Step II, we will test whether the changes in PHQ9 scores at week 4 through week 9 are statistically significantly different among the sertraline continuation arm, the mirtazapine augmentation arm and the mirtazapine switch arm. The null hypothesis that the changes are not different among the treatment arms will be tested by examining treatment effect parameters in the repeated measures analyses of all the eligible subjects in the ITT analysis. We will use random effects model taking into consideration the Step I randomization and Step II randomization factors. We will examine interaction effect of Step I cluster randomisation. The test will be double-sided. The alpha error is set at 0.05 and statistical power at 0.80. We will impute the missing data and carry out sensitivity analyses as necessary.

##### Secondary analyses

We will perform secondary analyses to supplement our primary analyses and to obtain more elaborate understanding of our clinical questions. The secondary analyses will use models similar to those of the primary analyses. We will calculate relative risks and their 95% confidence intervals for differences in proportions. We will calculate hazard ratios and their 95% confidence intervals for differences in treatment continuation.

Details of the statistical analyses will be laid down in the "Statistical Analysis Protocol", which will be prepared by the statistician in the Steering Committee by the time the analyses will be undertaken.

##### Interim analyses

We will not perform interim analyses to examine the study hypotheses for two reasons. First, we are not expecting a huge effect size for the planned comparisons and second, it is theoretically likely that trials stopped early for benefit may overestimate the true effect size.

However, we will examine the following aspects in order to ascertain the feasibility of the study without revealing the treatment allocation.

1) Number of entered subjects per trial site to calculate the number of trial sites and the time necessary to complete the intended study

2) The intra-cluster correlation coefficient will be calculated in order to make sure that it is not very different from the one assumed in this protocol. We will re-calculate the sample size if necessary.

The analyses for the pilot study will be given in detail in the "Statistical Analysis Plan."

### Ethical Aspects

#### Adherence to the study protocol and study manual

All the researchers participating in this trial will place the participants' safety and human rights above everything else and will adhere by the study protocol and the study manual so long as they do not undermine their safety and human rights.

#### Regulations to be adhered to

All the researchers participating in this trial will abide by the Declaration of Helsinki and its amendments as well as the Ethics Guideline for Clinical Research (2008 revision, Ministry of Health, Labour and Welfare).

#### Procedures for informed consent

Before entry into the trial, the trial physician must explain the following items using the written materials and make sure that the participant has understood the contents of the trial well. Written informed consent will only then be obtained from the participant.

1) About clinical trials

2) Objectives of the trial

3) Name and position of the principal trial physician and names of the participating trial physicians

4) Procedures and duration of the trial and what happens after the trial is over

5) Advantages to be expected and disadvantages to be anticipated

6) Other available treatment options

7) The participant can withdraw consent and stop participating in the trial at any time

8) There is no disadvantage if the subject does not participate in the trial or stops participating in the trial.

9) Medical records that are related to this trial will be seen by study personnel of this trial

10) Privacy will be maintained and protected

11) Contact address and method when the participant wants more information about the clinical trial or when he/she feels unwell

12) Compensation insurance for any health hazards

13) Fundings for this trial

14) Others

#### Protection of privacy

All the researchers and outsourcers of this trial must strictly protect personal information of the participants in adherence with the Ethics Guideline for Clinical Research and the Private Information Protection Law.

Each trial site, each regional centre and the national centre will collect information in anonymized and linkable format. The data centre will not deal with personal information of the participants. The linking information for the participants is strictly managed at each trial site or at the national centre without being computerized, i.e. in paper format.

At week 3, week 9 and week 25, the central rater will administer PHQ9 and FIBSER by telephone while being blind to the participant's allocated treatment. The central CRC will arrange this blinded telephone call by obtaining the participant's name and telephone number from each clinic every time. The central CRC will not keep this privacy information at the national centre office.

#### Approval by the IRB

This study has been approved by the Ethics Committee of Kyoto University Graduate School of Medicine, the Institutional Review Boards (IRBs) of Nagoya City University Hospital and of Kochi Medical School Hospital.

Each trial site will seek approval of the same protocol if it has its own IRB. If there is no IRB, the trial site will commission its approval to the IRB at Nagoya City University Hospital or at Kochi Medical School Hospital.

### Compensation Insurance

All the protocol interventions in the current trial are within the approved dosage and administration in Japan. However, because the trial involves random allocation, we will contract a private health insurance to compensate for health hazards that have arisen due to this trial. The contract will be between Kyoto University and Tokio Marine and Nichido Fire Insurance Company. This insurance will cover only death or grade 1 impairment or grade 2 impairment whose causal relationship with the trial cannot be negated. Grades 3-14 impairments will not be covered by this insurance but will be covered by the National Health Insurance, which therefore can incur some copayment. Because all the protocol interventions are within the approved dosage and administration, any health hazards may be object of the National Rescue Scheme for Side Effects. If there is any negligence on the part of the physician, it may be covered with the doctors' liability insurance.

### Study Organization

#### Steering Committee

The Steering Committee will hold online meetings every two weeks and offline meetings every two months.

Principal investigator:

Toshiaki A. Furukawa, MD, PhD (Kyoto University Graduate School of Medicine/School of Public Health, Department of Health Promotion and Human Beahvior)

Co-principal investigators:

Tatsuo Akechi, MD, PhD, Norio Watanabe, MD, PhD (Nagoya City University Graduate School of Medical Sciences, Department of Psychiatry and Cognitive-Behavioral Medicine)

Shinji Shimodera, MD, PhD (Kochi University Department of Neuropsychiatry)

Mitsuhiko Yamada, MD, PhD, Masatoshi Inagaki, MD, PhD (National Center for Neurology and Psychiatry, Institute of Mental Health)

Trial statistician:

Naohiro Yonemoto, MPH (National Center for Neurology and Psychiatry, Translational Medical Centre)

#### Data and Safety Monitoring Board: DSMB

DSMB will consist of three professionals in clinical trials and in psychiatry, who are not involved in this trial: Dr Teruhiko Higuchi (Psychiatrist, National Centre for Neurology and Psychiatry), Professor Yoshio Hirayasu (Psychiatrist, Yokohama City University) and Akiko Kada (Biostatistician, National Cerebral and Cardiovascular Center). The purpose of DSMB is to check the data monitoring reports prepared by the data centre and make recommendations, where necessary, to the principal investigator.

#### Research organization

**Figure 3:**
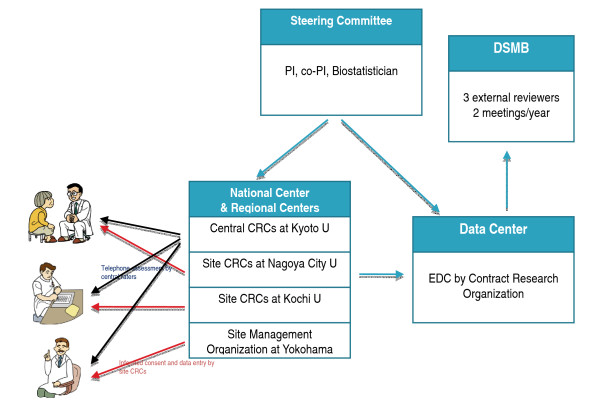
Organizational structure for the pilot study

Figure 3 shows the organizational structure for the pilot study.

#### Data centre

The data centre will be in charge of collecting and managing information independently from the researchers. The data centre will handle site registration, participant registration and allocation, monitor data entry, manage quality assurance of data, manage quality control of data, prepare monitoring reports, and prepare datasets for statistical analyses.

The data centre will make monitoring reports on the progress of recruitment, progress of data collection and adverse events to the Steering Committee and DSMB.

The data centre will be entrusted a contract research organization to be chosen by public tender bidding.

#### National centre and Regional centres

The national centre office will be located within Department of Health Promotion and Behavioral Medicine, Kyoto University School of Public Health. It will have a central rater, a CRC and a secretary.

There will be regional centres at Nagoya City University and Kochi University. There will be site CRCs who will make weekly visits to participating trial sites to assist informed consent procedures and to monitor and enter clinical data.

## Discussion

The SUN(^_^)D is an assessor-blinded, parallel-group, mutli-centre randomised controlled trial sequentially comparing active treatment options and combinations currently approved for treatment of depression in Japan.

The prominent characteristics of the SUN(^_^) D include the following.

First, the treatment arms in this trial are based on true clinical uncertainty, because all of them are treatment options currently approved by the regulatory bodies in Japan. In other words, they represent treatment alternatives from which both clinicians and their patients have difficulties choosing at the present moment.

Second, the clinical questions to be examined in this trial pertain to urgent and critical decision points for which the world psychiatric and psychopharmacological research community has hitherto failed to provide guidance or answer to.

Third, the trial will take place mostly in front-line psychiatric facilities such as private practices and departments of psychiatry of general hospitals from all over Japan, where many if not most of the patients with major depression receive their initial treatment. Because Japan does not have the primary care system as represented by the general practitioners in UK, these are the primary care mental health services in Japan and the current trial is thus expected to have maximum external validity with respect to initial treatment of major depression in Japan.

Fourth, several important measures are built in to ensure good internal validity for this pragmatic trial, such as central randomization, blind assessment of symptoms and side effects via telephone and adherence to true intention-to-treat principle through differentiation of discontinuation of protocol treatments and withdrawal from study. Whether we can achieve the last point will depend on whether we can follow up and assess all the patients even when they stop or deviate from the assigned treatments.

As discussed in the Introduction, major depression represents the greatest non-fatal burden of disease for the humankind, with commensurate rise in spending on the antidepressants world-wide. We maintain that this must be accompanied by commensurate increase in evidence base to guide their wise clinical administration. For example, the total annual sales of antidepressants amount to 120 billion yen (1.3 billion US dollars), and we would like to advocate that at least 0.1% of this sum be spent on public-domain pragmatic research of their use for mood and anxiety disorders. Many urgent and critical clinical questions can be answered with this research funds only if the research can be simple and pragmatic enough. We hope that SUN(^_^)D can be a template for such future clinical trials, and that it ultimately can provide good evidence to improve the treatment guidelines for depression in the world.

## List of abbreviations

5-HT: 5-hydroxytryptamine; BDI2: Beck Depression Inventory-II; C-CASA: Columbia Classification Algorithm of Suicide Assessment; CRC: Clinical Research Coordinator; CRO: Contract Research Organization; CYP: Cytochrome pigment; DALY: Disability-Adjusted Life Years; DSMB: Data Safety Monitoring Board; EDC: Electronic Data Capturing; FIBSER: Frequency, Intensity, and Burden of Side Effects Rating; MANGA: Meta-Analyses of New Generation Antidepressants; MAO: Monoamine oxidase; NICE: National Institute of Clinical Excellence; NaSSA: Noradrenergic and Specific Serotonergic Antidepressant; NNT: Number needed to treat; PHQ9: Personal Health Questionnaire-9; RCT: Randomized controlled trial; SMO: Site Management Organization; SNRI: Serotonin & Noradrenalin Reuptake Inhibitor; SSRI: Selective Serotonin Reuptake Inhibitor; STAR*D: Sequenced Treatment Alternatives to Relieve Depression; SUN ☺ D: Strategic Use of New generation antidepressants for Depression.

## Competing interests

TAF has received honoraria for speaking at CME meetings sponsored by Astellas, Dai-Nippon Sumitomo, Eli Lilly, GlaxoSmithKline, Janssen, Kyorin, MSD, Meiji, Otsuka, Pfizer, Shionogi and Yoshitomi. He is on advisory board for Sekisui Chemicals and Takeda Science Foundation. He has received royalties from Igaku-Shoin, Seiwa-Shoten, Nihon Bunka Kagaku-sha and American Psychiatric Publication. TA has received speaking fees and/or research funds from Astellas, Astra-Zeneca, Bristol-Meyers-Squib, Daiichi-Sankyo, Dainippon-Sumitomo, Eisai, GlaxoSmithKline, Janssen, Kyowa-Hakko-Kirin, Lilly, Meiji, Otsuka, Pfizer, Sanofi-Aventis, Shionogi and Yakult. NW has received speaking fees and/or research funds from Dainippon-Sumitomo, GlaxoSmithKline, Lilly, Otsuka, Pfizer, Asahi-Kasei and Shering-Plough. SS has received speaking fees and/or research funds from Astellas, Dainippon-Sumitomo, GlaxoSmithKline, Janssen, Lilly, Otsuka, Pfizer, Shering-Plough, Shionogi and Yoshitomi. MY and NY have no conflicts of interest to declare. NY received royalties from Seiwa-Shoten. MI has received a speaking fee from Lilly. KM has received speaking fees from Astellas, Dainippon-Sumitomo, GlaxoSmithKline, Janssen, Lilly, Meiji, Otsuka, Pfizer and Shering-Plough.

## Authors' contributions

TAF conceived the study, TAF, MY, MI and NY prepared the original manuscript. TAF, TA, SS, MY, KM, NW, MI and NY participated in the refinement of the protocol. NY is the trial statistician. All authors critically reviewed and approved the final version of the manuscript.
